# The Topical Effect of Grape Seed Extract 2% Cream on Surgery Wound Healing

**DOI:** 10.5539/gjhs.v7n3p52

**Published:** 2014-10-28

**Authors:** Ali Asghar Hemmati, Maryam foroozan, Gholamreza Houshmand, Zahra Beigom Moosavi, Mohammad Bahadoram, Nader Shakiba Maram

**Affiliations:** 1 Department of Pharmacology and Toxicology, Herbal Centre, Pharmacy School, Ahvaz Jundishapur University of Medical Sciences, Ahvaz, Iran; 2 Department of Dermatology, Ahvaz Jundishapur University of Medical Sciences, Ahvaz, Iran; 3 Medical Student Research Committee, Ahvaz Jundishapur University of Medical Sciences, Ahvaz, Iran; 4 Department of Pharmaceutics, Pharmacy School, Ahvaz Jundishapur University of Medical Sciences, Ahvaz, Iran

**Keywords:** grape seed extract, skin cream, wound healing

## Abstract

**Background:**

Reducing the wound healing time is crucial in wound as it lowers the chance of infection and decreases complications and cost. Grape seed extract has the ability to release endothelial growth factor and its topical application results in contraction and closure of the skin wound. Furthermore, it possesses antioxidant and antibacterial properties. In several studies it has been proved effective in animals. Therefore, due to low side effects and recognition of herbal medicine, we decided to evaluate the effect of grape seed extract 2% herbal cream on human skin lesions.

**Materials:**

This study is a double blind clinical trial conducted on two groups of treatment and placebo. Surgery was performed on skin lesions such as skin tags and moles which were found on the neck, trunk and limbs (except for face). After enrollment and obtaining informed consent from participants, they were randomized into two groups of treatment and placebo. Excision of the lesions was done by surgical scissors. The lesions got restored by secondary intention method. After the first day of treatment, the patients were visited on the 3rd, 7th, 10th, 14th, and 21st day. Grape seed extract cream 2% was produced and coded by the Faculty of Pharmacy, Ahvaz University of Medical Sciences. In order to compare the two groups, T-test was used. For time assessing, analysis of variance with repeated measures was employed.

**Results:**

The results showed complete repair of wounds averagely on day 8 for the treatment group and on day 14 for the placebo group, which was clearly significant in terms of statistical difference (p=0.00).

**Conclusion:**

Proanthocyanidins in grape seed extract trigger the release of vascular endothelial growth factor and its topical application causes wound contraction and closure. Curing skin lesions with grape seed extract caused proliferation areas with protected boundaries in epithelium, increased cell density and increased deposition of connective tissue at the wound site which in general improves cellular structure in wound. In addition, its anti-inflammatory and anti-microbial properties are effective in wound healing.

## 1. Introduction

The skin is a barrier against environmental factors and losing parts of it can cause disability or even death. Wound healing is a complex and dynamic process with multiple phases. Several local and systemic factors such as age, certain medications (corticosteroids, anticoagulants, aspirin), nutrition (protein, vitamins specially A & C, zinc, iron), circulation and tissue hypoxia status, localized compounds (antibiotics, antiseptics) and wound dressing affect the healing process. Achieving faster recovery and less scarring is still one of medicine’s most important goals. Moreover, shortening recovery time is of critical importance since it reduces the chance of infection and decreases complications and costs.

Recent studies suggest that the use of plant resources alone or in combination with chemical agents is of beneficial value in wound healing. Grape seed extract is rich in powerful antioxidant compounds such as Proanthocyanidin and Polyphenol. Proanthocyanidin effect in the body is 20 times more than vitamin C and 50 times more than vitamin E. By neutralizing the impact of free radicals these antioxidant compounds prevent cell damage caused by free radicals. For this reason grape seed extract is used in the treatment of disorders associated with increased free radicals. It is reported that grape seed possesses good preventive effects against oxidative damage to DNA (Nassiri-Asl & Hosseinzadeh, 2009). Other effects have been cited in several studies including anti-carcinogenic effects, protection of the body against sun damage, improving vision, improving flexibility in joints, arteries and tissues such as the heart, improving circulation by strengthening capillaries, arteries and veins and reducing blood pressure (Shi, Yu, Pohorly, & Kakuda, 2003; Nassiri-Asl & Hosseinzadeh, 2009; Feringa, Laskey, Dickson & Coleman, 2011). Antimicrobial and anti-viral effects have been derived from different components of grape; among them are gallic acid, hydroxyl cinnamic acid, flavonols, trans-resveratrol and tannins. Also anti-listeria activity has been reported in grape seed 1% extract (Al-Habib, Al-Saleh, Safer, & Afzal, 2010; Kao et al., 2010). Although several studies have revealed the effectiveness of grape seed extract in wound healing in animals (Diegelmann & Evans, 2004; Nayak, Ramdath et al., 2010; Hemmati, Aghel et al. 2011; Nayak et al., 2011), no study has been done on humans so far. In this study we investigated the topical effect of grape seed extract on skin wound healing in humans.

## 2. Methods

### 2.1 Study Population

This double blind clinical trial was conducted over a 3-week-period on patients admitted to Imam Khumeini hospital, Ahvaz Jundishapur University of Medical Sciences, Ahvaz, Iran, dermatology clinic for surgery on skin lesions. The protocol of the trial was also registered to the Iranian Registry of Clinical Trials (IRCT) under the number IRCT2014110314190N6 and has permission for implementation from the ethics committee of Ahvaz Jundishapur University of Medical Sciences.

Inclusion criteria comprised skin lesions such as skin tag and mole on the neck, trunk and limbs (except for face), age between 14 and 50 years, clinical benignity of the lesion, size of the lesion larger than 3mm and smaller than 1cm. For exclusion criteria the following were considered; having any underlying diseases, immunosuppressants or other certain medications, pregnancy or lactation, smoking or any long-term drug use, and having concurrent malignancy.

### 2.2 Study Intervention

A total of 40 patients were enrolled based on the inclusion criteria. After obtaining informed consent from patients according to GCP principles, Participants were assigned to one of the two groups based on the table of random numbers either to grape seed 2% cream (20 patients) or placebo (20 patients).

All the surgical procedures were performed by a dermatology resident. Removal of the lesion was done after numbness (with Lidocaine) and sterilization (using Betadine) assisted by means of a scalpel using surgical scissors method. Lesions were treated with secondary intention method. Once hemostasis achieved, the area of the lesion was measured. Subsequently, cream was applied (either grape seed or placebo) on the wound and the wound was dressed. Topical cream of grape seed extract was produced and coded by the Faculty of Pharmacy, Ahvaz University of Medical Sciences, Ahvaz, Iran. Grape seed 2% cream is based on eucerin and the aqueous phase in it, is greater than the fat phase (about 60%). In the formulation of this medication the extract of red grape seed and preservatives are used. Also specific compounds are used in order to sustain and stabilize the color and flavor of it. Certain agents were employed to homogenize the mixture of the two phases (water & fat). The placebo cream, except for the effective compound (grape seed), is similar to the original one. All thermal, shelf-life, high speed centrifuges, biological testing and ensuring the non-separation of water-fat phase of the drugs used in this study were fully accomplished in the pharmaceutical technology development center, Faculty of pharmacy, Ahvaz Jundishapur University of Medical Sciences. Instructions on application of the cream were given to the patients the following days, so as to after washing the hands and location of the wound, the patient should apply the cream on the surface of the wound twice daily and each time to an extent enough to cover the wound area (half a knuckle). Then the patients were visited on the 3rd, 7th, 10th, 14th, and 21st day. In all the patients, transparent sheet was used to measure the surface area of the sample and the shape of the lesion was graphed. Furthermore, in every visit the patients’ wounds were photographed with an 8 MP Cannon camera. On the first day, wound level was considered 100% and improvement 0%, the next days, wound level and the rate of improvement were assessed and registered. Patients’ data including demographic data, skin type, location of lesion, clinical diagnosis of lesion and lesion size were collected with the use of a questionnaire.

### 2.3 Statistical Analysis

At the end of the study, statistical analysis was done by using T-test and repeated measures with significant level at p<0.05.

## 3. Results

Two cases from the grape seed group and three from placebo withdrew from the study which was not statistically significant. The data of the 35 patients who completed the study was analyzed at the end of the trial. Both groups were matched for age and sex. Other characteristics of the two groups had no statistically significant difference in the beginning of the study (p> 0.05) (Table 1).

**Table 1:** Demographic characteristics of the study population

variable		Placebo group N (%)	Treatment group N (%)	P
sex	male	5 (27.8)	5 (29.4)	0.5
	female	13 (72.2)	12 (70.6)	
Skin type	Level2	4 (22.2)	4 (23.5)	
	Level3	13 (72.2)	12 (70.6)	0.9
	Level4	1 (5.6)	1 (5.9)	
Lesion location	neck	24 (75)	24 (77.4)	
	armpit	5 (15.6)	5 (16)	0.07
	thigh	3 (9.4)	2 (6.6)	
age	mean	30.0 ±5.68	30.3 ±5.75	0.8
	min	22	21	
	max	45	47	

The results of the measurement of lesions in both groups before treatment showed that there was no statistically significant difference pertaining the mean wound surface area (p=0.57) (Table 2).

**Table 2:** The mean wound surface area in the two groups at baseline

variable	n	Mean wound surface area (mm2)	Standard deviation	P
Placebo group	32	9.09	8.41	0.57
Treatment group	31	7.88	7.74	

Comparison of wound healing in both groups suggests significant difference between the two groups on day 3 (p=0.0001), 7 (p=0.0001), 10 (p=0.0001) and 14 (p=0.036).

**Figure 1:**
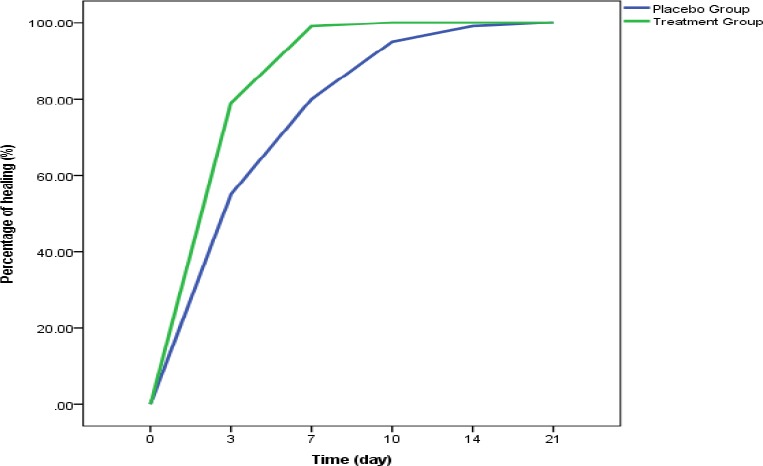
Comparison of mean percentage of wound healing improvement on different days after treatment in the two groups of grape seed and placebo

On average, lesions of the grape seed group showed full recovery on day 8 (8.06 ± 1.45) and the placebo group on day 14 (14.40 ± 3.94) which is clearly significant in terms of statistical comparison (p=0.0001).

One to 2 lesions of each patient was included in the study. From the treatment group, 31 and from the control, 32 lesions were examined. Three days into the treatment, neither of the two groups displayed complete healing. While the placebo group had no recovery on the 7^th^ day, 20 (64.5%) out of 31 cases in the grape seed group reported full recovery which was statistically significant (p=0.0001).

Follow up of patients’ improvement report revealed that on day 10, in the placebo group 9 cases (28.15%) displayed complete healing, whereas in the grape seed group all 31 cases equivalent for 100% had made full recovered. This statistical difference is also clearly significant (p=0.0001). Despite the complete healing of all cases of the grape seed group on day 10, follow up continued on the placebo group. Results show that on day 14^th^ after treating 25 cases (78.1%) and on day 21^st^ since the initiation of the treatment all 32 cases in the placebo group had healed completely.

**Figure 2:**
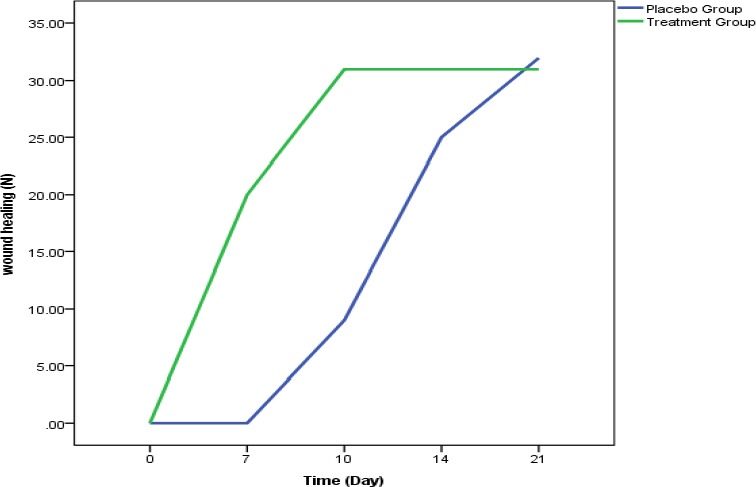
Comparison of the number of wounds healed in both groups on different days after the start of the treatment in the two groups of grape seed and placebo

**Figure 3:**
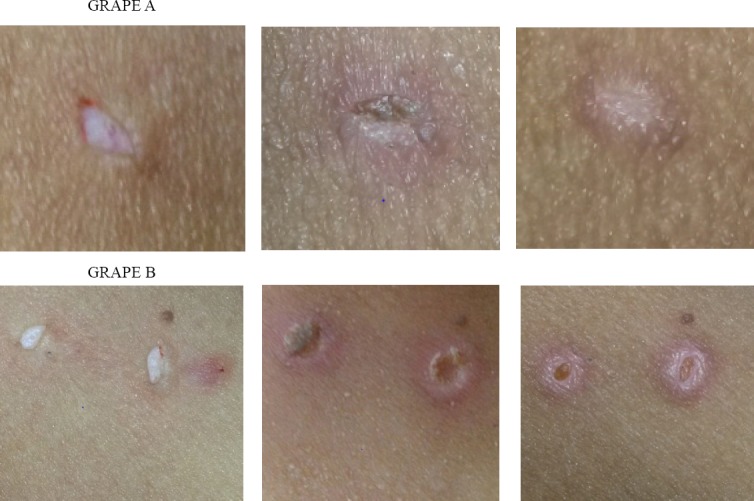
The rate of wound healing on the first, third and seventh day of the study in grape seed group (A) and placebo (B). Note that picture no.3 belongs to day 10

## 4. Discussion

The use of plants and plant products for wound healing has a long history. Especially in countries like India, China and Iran that there is more interest in traditional medicine, valuable information is available on using unknown plants for treating wounds (Feringa et al., 2011). However, little scientific research has been done to prove their benefits. Today, the use of various herbal extracts that have a long history of use in traditional medicine, has also been considered in accelerating skin wound healing (Bhatia et al., 2010; Li et al., 2012). Extensive studies suggest that grape seed extract has health benefits in many ways as a result of its antioxidant effect.

Grape seed extract is rich in flavonoids and the main flavonoids found in grape seed are procyanidolic oligomers (also called proanthocyanidins) which have antioxidant properties. Scientists believe phytochemicals are able to protect cells against the damaging effect of unstable oxygen and thus prevent and inhibit occurrence of disease (Bhatia et al., 2010). Proanthocyanidins are a subtype of flavonoids and their anti-oxidant properties in wound healing has been proved. Grape seed extract is a great source of proanthocyanidins, thereby according to the results of this clinical trial, we can probably say that proanthocyanidins found in grape seed along with other flavonoids, can play an important part in skin wound healing process.

Grape seed has been reported to have good preventive effects against oxidative damage to DNA (Nassiri-Asl & Hosseinzadeh 2009). Grape seed proanthocyanidins have antioxidant power 20 times more than vitamin C and 50 times more than vitamin E. However, it has an insignificant effect on the body stores of vitamin E and C (Shi et al., 2003). Studies suggest that grape seed proanthocyanidins help protect the body against tissue damage (Shi Shi et al., 2003; Nassiri-Asl & Hosseinzadeh 2009). Antimicrobial and anti-viral effects have been derived from different components of grape (Su & D’Souza, 2011). Also several studies have revealed the effect of grape seed extract and its mechanism on wound healing in animals (Diegelmann & Evans, 2004; Nayak et al., 2010; Hemmati et al., 2011; Shivananda et al., 2011).

In this study, the topical effect of grape seed extract was studied on human skin wound healing. The results of this study demonstrated that after 3 days of treatment, the wound size in the group receiving grape seed extract clearly diminished compared to the placebo group. On the seventh day of the study in spite of non-cure of the entire placebo group, in the group receiving grape seed extract, 20 cases out of 31 (64.5%) had fully recovered and only slight erythema was evident at the wound site. Also on the 10^th^ day of the study, all lesions in the group receiving grape seed extract had fully restored, whereas only 9 patients in the placebo group (28.15%) had full restoration.

Following-up on the placebo group, on the 14^th^ day, 25 cases (78.1%) and on the 21st day, 100% of lesions had full repair which was clearly significant in terms of statistical difference. In order to evaluate more precisely, we also examined the level of healing. It was revealed on day 3rd, the mean level of healing in the placebo group was 55.5% while this figure for the recipients of grape geed extract group was 79.44% which rised to 99.35% on the 7^th^ day versus 79.68 percent in the placebo group and finally the percentage of healing in the recipient group of grape seed extract after 10 days was 100% whereas in the placebo group about 4 percent of lesions had not complete healing (in the placebo group on day 10, the level of wound healing was 95.48%). The difference was clearly significant for wound healing in grape seed extract group as opposed to the placebo group which is consistent with the results from previous studies in this field.

In a study, Hemmati et al. (2011) showed that using grape seed extract 2% improves and accelerates the process of contraction and closure of wounds, shortening the healing time of 20 days in the placebo group to 13 days in the grape seed group. Also in shivananda nayak study the powder of grape peel was applied in healing the full-thickness of skin lesions in rats versus petrolatum and mupirocin, in which the contraction of wound was 100% in the grape peel group and full recovery achieved on day 13 since the start of the treatment (Nayak et al., 2010). The results of our study showed that on average, the lesions of the treatment group reached full recovery on day 8 (8.06 ± 1.45) and in the placebo group on day 14 (14.40± 3.94) which was statistically significant.

The results of our examinations revealed better improvement which is probably due to better wound healing in humans or superficiality of the wounds. Hemmati et al. (2011) found that the process of recovery and healing effects of grape seed extract 2% a day after the start of treatment was clearly evident. Also in this study the effect of grape seed extract 2% was evidently greater than phenytoin in wound healing. However, high concentrations of grape seed extract did not produce better results than grape seed extract 2% (Hemmati et al., 2011).

The results of this study showed than mean healing time in the grape seed group was 8 days versus 14.4 days in the placebo group. Grape seed extract has the ability to release endothelial growth factors and its topical use causes wound contraction which is an important part of the natural healing process. Curing skin lesions with grape seed extract caused proliferation areas with protected boundaries in epithelium, increased cell density and increased deposition of connective tissue at the wound site which in general improves cellular structure in wound (Khanna et al., 2002; Diegelmann & Evans, 2004).

## 5. Conclusion

Grape seed extract proanthocyanidins in addition to anti-oxidant properties, may trigger the release of vascular endothelial growth factor along with the promotion of fibroblasts to produce more collagen fibres. Therefore and its topical application causes wound contraction and closure. Curing skin lesions with grape seed extract causes proliferation areas with protected boundaries in epithelium, increased cell density and increased deposition of connective tissue at the wound site which in general improves cellular structure in wound. Furthermore, its anti-inflammatory and anti-microbial properties are effective in wound healing. Based on the data from this study and previous studies we may suggest the clinical use of tropical formulation of grape seed extract 2%.
